# 3D Printing of Temporary Prostheses for Controlled-Release of Drugs: Design, Physical Characterization and Preliminary Studies

**DOI:** 10.3390/ph14121240

**Published:** 2021-11-29

**Authors:** Carlos Bueno-López, Carlos Tamarit-Martínez, Adrián M. Alambiaga-Caravaca, Cristina Balaguer-Fernández, Virginia Merino, Alicia López-Castellano, Vicent Rodilla

**Affiliations:** 1 Instituto de Ciencias Biomédicas, Departamento de Farmacia, Facultad de Ciencias de la Salud, Universidad Cardenal Herrera-CEU, CEU Universities, C/Santiago Ramón y Cajal s/n, 46115 Valencia, Spain; car.bueno.ce@ceindo.ceu.es (C.B.-L.); car.tamarit.ce@ceindo.ceu.es (C.T.-M.); adrian.alambiagacaravaca@uchceu.es (A.M.A.-C.); cbalaguer@uchceu.es (C.B.-F.); 2 Departamento de Farmacia y Tecnología Farmacéutica y Parasitología, Facultad de Farmacia, Universitat de València, Av. Vicente Andrés Estellés s/n, 46100 Valencia, Spain; virginia.merino@uv.es; 3 Instituto Interuniversitario de Investigación de Reconocimiento Molecular y Desarrollo Tecnológico (IDM), Universitat Politècnica de València, Universitat de València, 46005 Valencia, Spain

**Keywords:** 3D printing, fused deposition modelling, FDM, controlled-release, medical devices, personalized prosthesis, polylactic acid, PLA

## Abstract

In recent years, the use of 3D printing technologies in orthopedic surgery has markedly increased, as they offer the possibility of printing personalized prostheses. The work presented in this article is a preliminary study of a research project which aims to manufacture customized spacers containing antibiotics for use in joint replacement surgery. The objective of this work was to design and print different 3D constructs to evaluate the use of different materials, their properties after the process of 3D printing, such as resistance, and the release kinetics of drugs from the constructs. Different designs and different materials were analyzed to obtain a 3D construct with suitable properties. Our design takes advantage of the micropores created between the layers of the 3D printed filaments to release the contained drug. Using polylactic acid (PLA) we were able to print cylindrical structures with interconnected micropores and a hollow chamber capable of releasing methylene blue, which was selected as a model drug. The final PLA 3D construct was printed with a 10% infill. The physical and technological characteristics, morphological changes at body temperature and interaction with water were considered to be acceptable. The PLA 3D printed constructs were found to have sufficient strength to withstand a force of 500 kg. The results obtained allow to continue research in this project, with the aim of manufacturing prostheses containing a reservoir of antibiotics or other drugs in their interior for their subsequent controlled release.

## 1. Introduction

Infections are among the most serious complications of arthroplasty interventions, both for patients and surgeons. In most cases, the infection-causing bacteria grow adhered to the surface of the implant as biofilms [1,2]. These infections are difficult to treat because the biofilm protects bacteria against the host’s immune system and also protect them against the effects of systemically administered antibiotics [1,3]. In the first two years after surgery, periprosthetic hip infections have been estimated to be between 0.3 and 1.7%, whereas in knee arthroplasties the percentage of infections would affect 0.8 to 1.9% of patients [4,5,6,7]. When such an infection occurs, it has serious adverse effects on the patient and a substantial economic impact which results in an increased health care burden, because the prosthesis needs to be removed, implementing repeated surgical procedures and long-term treatment with antibiotics [8,9]. A two-stage revision arthroplasty has been the preferred treatment for periprosthetic infections, a procedure first described in 1983 [10]. A two-stage revision arthroplasty involves removing the infected prosthesis, which is the source of pathogenic bacteria and implementing an antibiotic treatment, either systemically and/or locally by impregnating it in a static or an articulated bone cement (poly-methyl-methacrylate or PMMA) spacer, which may be handmade or custom-moulded/prefabricated [2,10]. At times, other materials such as metal and polyethylene can be used for the spacers and these can also be combined with cement [10]. Once the infection has been eradicated, in the second stage, a new prosthesis is implanted [10,11]. The role of spacers in these interventions is mainly to maintain soft tissue tension and to avoid tissue retraction, while maintaining the joint space to facilitate reimplantation during the second stage [12]. However, the use of a spacer is not problem-free. The most common complications associated with their use are tilting, mediolateral translation, dislocation, subluxation and fractures [10,13]. 

Combining antibiotics with cement has been successfully used since the 1990s when its use and effectivity was first reported. However, neither the time-scale of antibiotic release from the bone cement nor the amount of antibiotic released are fully understood. There are conflicting reports in the literature as some authors report that the release of the antibiotic lasts a few days, whereas others maintain that antibiotic release only takes place in the first couple of days or so; to complicate this issue further there is a another group of authors reporting that antibiotic release only happens in the first few hours after implantation [10,14,15]. Other problems related to the use of these antibiotic-loaded bone cements is the possible modification of the mechanical properties of the cement because the addition of antibiotics weakens its structure [16]. Heat-sensitive antibiotics may suffer alteration/deterioration of their properties due to the exothermic reactions taking place during polymerization of cement. Furthermore, high dose of antibiotics being released locally on osteoblasts has been reported to cause toxicity [10,17] and when aminoglycosides are used there is the possibility of acute renal failure if the antibiotic enters the systemic circulation in large doses [18]. 

Three-dimensional (3D) printing has evolved very rapidly over the last few years and has entered the medical field [19,20], particularly being used for education [21], simulation [22], pre-operative planning [23,24] and implantation [24]. The use of a 3D-printed temporal prosthesis (custom-made from computerized tomography (CT) images or magnetic resonance imaging (MRI)), for patients undergoing two-stage arthroplasties would provide an articulated spacer perfectly suited for the anatomy of each patient. If antibiotics directed to the specific pathogens causing the infection are incorporated to the temporal prosthesis to be released at local level, the efficacy of treatment would be greatly improved, while maintaining the anatomical space at the site of infection.

There are many widely used techniques used for 3D printing such as stereolithography (SLA) commonly called, resin 3D printing or selective laser sintering (SLS). SLA uses a light source (a laser or projector) to cure liquid resin and transform it into hardened plastic, whereas SLS is a powder bed printing technology that uses a laser to join tiny bits of nylon powder, tracing the geometry layer by layer and working from the bottom of the part upwards [25].

**Figure 1:**
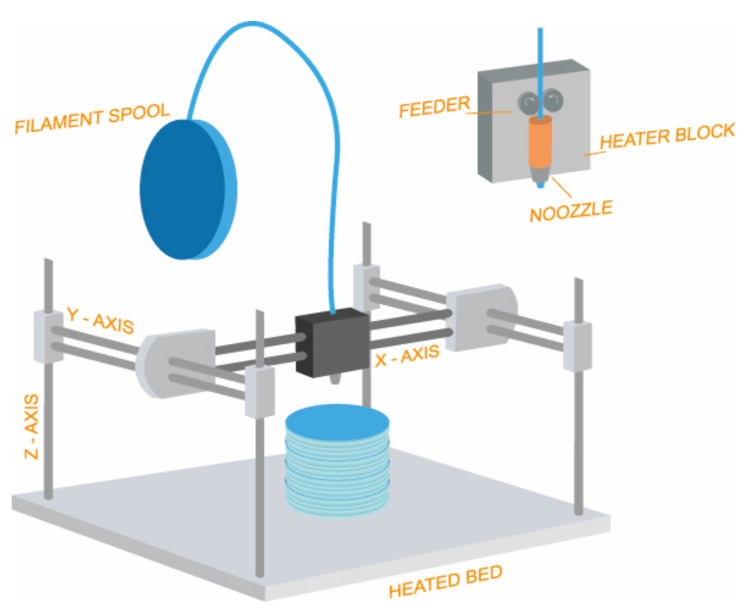
Schematic representation of fused deposition modeling (FDM).

Another widely used technique in 3D printing is fused deposition modelling (FDM), which is based on solid base additive manufacturing technology (3D printing). It involves the use of thermoplastic materials supplied in the form of filaments that are extruded through a nozzle under defined pressure conditions and progressively layered in a melted/softened state to build up the final product. The material is previously fused in the heater block and pushed through the nozzle that moves to the correct horizontal position calculated with coordinates using two electric motors one for each axis (X and Y), depositing the material in the printer bed where it cools (Figure 1). Once a layer is printed the printing head is moved upwards with a third electrical motor, allowing the extrusion of the next layer which will fuse with the previously printed one [26]. Depending on the software and the printer used, quite a large number of parameters can be modified. 

Several materials can be used for FDM printing and many have great potential for biomedical applications. The mechanical properties of structures printed with them are also dependent on the pore architecture, pore size and porosity which can be readily designed and fabricated with the FDM technique [27]. FDM was selected for this work as it showed promise in the possibility of controlling the porosity of the final result throughout the combination of parameters during the 3D printing process.

There are several impact resistant, biocompatible materials such as poly-(lactic acid) (PLA), acronitrile butadiene styrene (ABS), polyethylene glycol terephthalate (PET-G) and polypropylene (PP) which can be printed using FDM technology. Some materials are commercialized for medical applications (e.g., ABS Medical Smartfil^®^) and have been shown to fulfil USP Class VI or ISO 10993-1 (Biological evaluation of medical devices) [28].

Polypropylene (PP) is a thermoplastic polymer that was discovered in 1954 by Giulio Natta, which was mass-manufactured as a polymer by the Italian firm Montecatini in 1957. PP is tough, light, flexible, food safe and has a range of properties that make it suitable for use in the manufacture of health care products [29,30]. Until recently, PP was not available for use in 3D printing applications and needed a special manufacturing technique: melt electrospinning writing. This technique was used to make nanofibers of polymers by electrospinning nanoparticles mixed with polymers producing nanofibers [29]. This technique was essential for accurate three-dimensional fabrication; however, nowadays is usable without it. 

PET-G is a high-strength material that withstands high-temperatures required for 3D printing with potential for applications such as medical devices as it complies with the international biocompatibility standard ISO 10993. From a technical point of view, PET-G seems to combine ABS resistance and ductility with PLA ease of printing. It also was found to be the 3D printed material that most accurately represented visual, tactile and other kinesthetics properties of human bones [31].

ABS plastic or Acrylonitrile Butadiene Styrene is an amorphous, impact-resistant, opaque thermoplastic that is widely used in the plastic industry. ABS is considered structurally very strong. This makes it an ideal choice for various applications that need strong and stiff plastic that is resistant to external strength impacts. It is highly used in applications such as protective housings, camera housings and stiff packagings which need to be structurally sturdy. ABS is considered relatively non-toxic and hence a harmless thermoplastic. Adverse health effects as a result of exposure to ABS plastic have yet to be reported. It contains no known carcinogens, does not leach and is stable [32].

Polylactic Acid (PLA) is a polymer approved by the FDA (Food and Drug Administration, Silver Spring, MD, USA) which has been used in a number of surgical devices. FDA has catalogued PLA as GRAS (Generally Regarded As Safe). PLA is heat resistant (it can withstand temperatures of 110 °C). Its biodegradation generates organic lactic acid, making PLA non-toxic to the human body [33]. Furthermore, in 2015 Weisman and co-workers were able to manufacture PLA filaments impregnated with gentamicin sulphate or methotrexate and showed that after extrusion the filaments had antimicrobial or chemotherapeutic properties [34]. Recently the same authors have printed catheters and demonstrated that a sustained release of these drugs can be maintained [28] making it therefore an optimal material for the purpose of our study. 

The work presented in this article corresponds to a preliminary study of a research project which aims to manufacture custom spacers containing antibiotics for their use in joint replacement surgical interventions. The objective of this work was to design and print different 3D constructs to be eventually used as temporary prostheses (spacers), as well as to evaluate the use of different materials and their physical properties after the process of 3D printing and characterize the release kinetics of substances included in the constructs.

## 2. Results and Discussion

### 2.1. Design of the Spacers 

**Figure 2:**
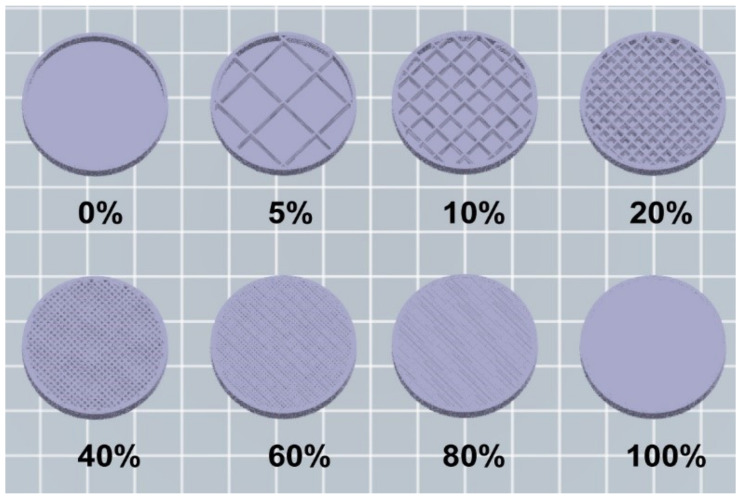
Visual representation of different infill percentage (0, 5, 10, 20, 40, 60, 80 and 100%) created with the program “Flashprint”.

Although there are some basic parameters which are shared through all the FDM systems, depending on the software and the printer used, several of parameters can be modified: (a) nozzle diameter, which reflects the diameter of the strand that will be deposited on the printer bed; (b) the feeding rate, which will define the thickness of the layer; (c) the printing speed; (d) the infill percentage, which represents the density of the construct controlling the size of the scaffold: an infill percentage of 100% is a printed solid object, while a 0% infill will represent a construct with no internal scaffolding (Figure 2).

Various designs were made to develop an optimal structural shape for both resistance and release of the drugs. Table 1 will serve to illustrate the different design iterations of the 3D constructs that were tested. As first approach to check the drug release from the inside, a solution of methylene blue (1 mg/mL) was introduced in the printed 3D constructs designs to rapidly observe if the release was taking place.

### 2.2. Material Strength Assessment

All 3D printed constructs were tested in both vertical and horizontal positions to evaluate the load they were able to support. The resistance was tested with the different materials printed with design VI, on the Zwick/Roell Z005 dynamometer. If this force was applied on a structure without breaking it, then it was considered an overload. When the behavior of horizontally-placed 3D printed constructs was studied, differences in resistance were noted due to elasticity of the materials.

Figure 2 shows the deformation (mm) as a function of the force applied (Kg). The test was performed with two limits: the test automatically stopped when 5% strength was lost and the other when the maximum deformation was 15% of the total value of the 3D construct. It was considered that if the material suffered a deformation greater than 15% the performance of a spacer would not be adequate. 

Regarding the material behavior, polypropylene (PP) shows high malleability, easily deforming under pressure. From our point of view this is perceived as a material weakness, because the 15% deformation limit is rapidly achieved without breaking and even when low forces have been applied. The results obtained with PP are less consistent than other materials and have a variety of weakness in layer adhesion and general adhesion to the bed of the printer as could be observed throughout the print process of our cylinders. Furthermore, even though PP shows clinical benefits, such as its flexibility and tenacity, very useful in operations like hernia repairs and pelvic organ prolapse surgery [35,36], recent studies show that implants made with this material might cause a reaction in the body’s immunologic system [30] and therefore we are concerned about possible implications derived from even a short term exposure to this material.

**Figure 3:**
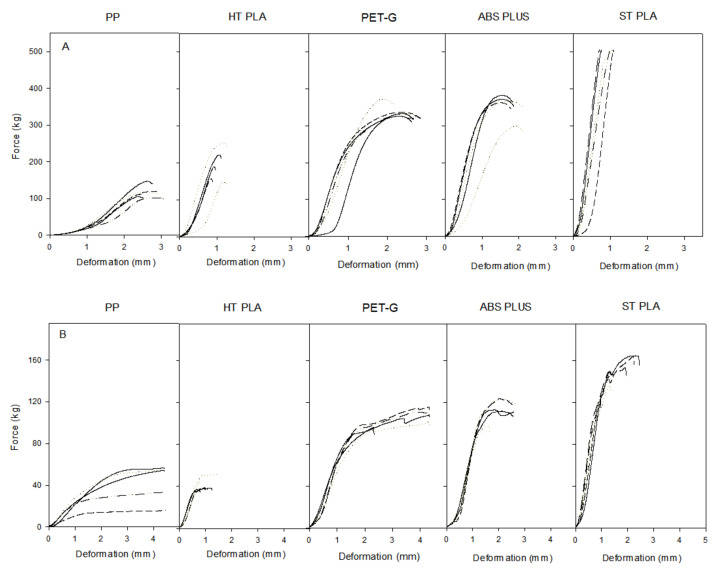
Representation of strength–stress test of the spacers in vertical (**A**) and horizontal (**B**) position for each material assessed (*n* = 5) at Zwick/Roell Z005 dynamometer.

Results obtained in our experiments with high temperature PLA (Ht PLA) did not meet the expectations of the material, evidencing the importance of selecting not only the type of material, but also checking its mechanical characteristics depending on the type of manufacturer/product. Even though Ht PLA endured a higher force than PP in the vertical position, it proved to be rather weak in the horizontal position: as it is a fragile material it breaks very easily. This can be deducted from the slope and the lack of deformation (Figure 3B). Furthermore, the material shattered making it unsuitable for a spacer.

Although PET-G showed much promise [31], results have not been able to match expectations, because although printability is far superior to other materials, resistance is not. Similarly even though in the literature ABS is praised for its performance when confronted by both dynamic and static loads [32], the same cannot be said about ABS 3D constructs. Results show that strength after 3D printing is lower than expected from the specification provided by the manufacturer. This might be due to poor layering adhesion as ABS is known to be a difficult-to-print plastic.

PET-G and ABS had both very similar breaking points, differing mostly in their behavior before breaking: while PET-G deformed before its rupture, ABS maintained its shape until breaking point (Figure 3, PET-G, ABS PLUS).

Finally, the material that presented greater resistance was the Standard PLA (St PLA). It supported the maximum load exerted by the dynamometer (500 kg) and therefore reached the point of overload in the vertical position (Figure 3A, ST PLA) It also achieved the highest breaking load in the horizontal position compared to the other materials, confirming its strength (Figure 3B). 

Further studies have shown that PLA degradation was found to be acceptable as molecular weight variation was proven to be only a 16% loss after 3 weeks of hydrolysis at 50 °C [33].

Table 2 and Table 3 show the values of breaking load, compression and percentage of compression of the cylinders vertically (Table 2) and horizontally (Table 3) for every material assessed.

Compression in the horizontal position was greater than in the vertical position (Table 2 and Table 3); this is due to the cylindrical shape of the 3D construct, which offers less resistance to compression when placed horizontally. It is also striking that Ht PLA does not increase its deformation in a horizontal position due to its brittleness. The large difference in strength between the vertical and horizontal positions is due to the lack of support of the outer layers on each other when force is exerted on the cylinder height.

The mechanical properties of St PLA were statistically compared with all other materials. Differences were found with all other materials (*p* < 0.05) in at least one of the comparisons carried out (breaking load and compression).

The material selected to carry out further analysis about the influence of other factors on the strength of the design and the material was St PLA since it was the most resistant and the literature review has shown the PLA has great biocompatibility.

**Figure 4:**
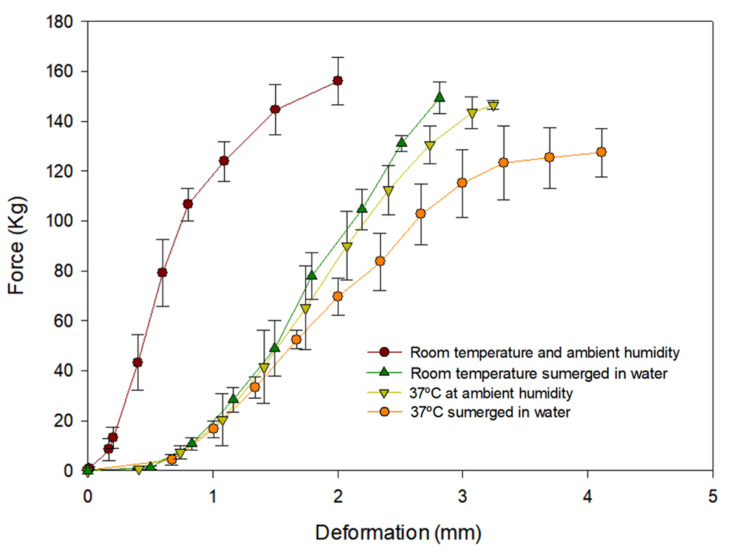
Graphic of strength–deformation test of the St PLA 3D constructs in horizontal position for each condition assessed (*n* = 5).

Figure 4 shows the difference in deformation and strength presented by the St PLA horizontally under different conditions. Conditions were chosen for the experiments based on the environment of the human body which the 3D constructs would eventually have to endure. The study was carried out in a horizontal position to observe better any weaknesses created by the different conditions as the lack of breaking in vertical position would not be able to provide such information. These data were obtained from the J.BOT dynamometer except at room temperature and ambient humidity because the first dynamometer was not able to achieve breakage, so it was repeated in the Zwick/Roell Z005 dynamometer.

Results show that both immersing the contrast in water and increase in temperature impact negatively on the strength of the 3D constructs. Furthermore, the effect increases when both conditions are combined. Moreover, deformation before breaking is the most affected parameter by environment conditions: submerging the construct in water at 37 °C results in 50% higher deformation than when the construct is at room temperature and ambient humidity.

Nevertheless, all results have an acceptable value for the use of St PLA in the spacers supporting the continuation of the studies.

### 2.3. Influence of Infill on Strength 

This study was carried out to evaluate how the infill percentage affects the resistance of the constructs. Our aim was to decrease the percentage of the infill as much as possible to maintain the maximal volume available for the drug reservoir as well as decreasing the printing time and achieving lower production costs.

Resistance tests were carried out using different infills (0, 2, 4, 6, 8 and 10%) until overload was reached in a vertical position (Figure 4). It is important to note that the infill type had overlapping beams (design VI) as the use of other types of infill, such as a honeycomb, could improve the resistance but worsen the diffusion as the previously described tests demonstrated.

**Figure 5:**
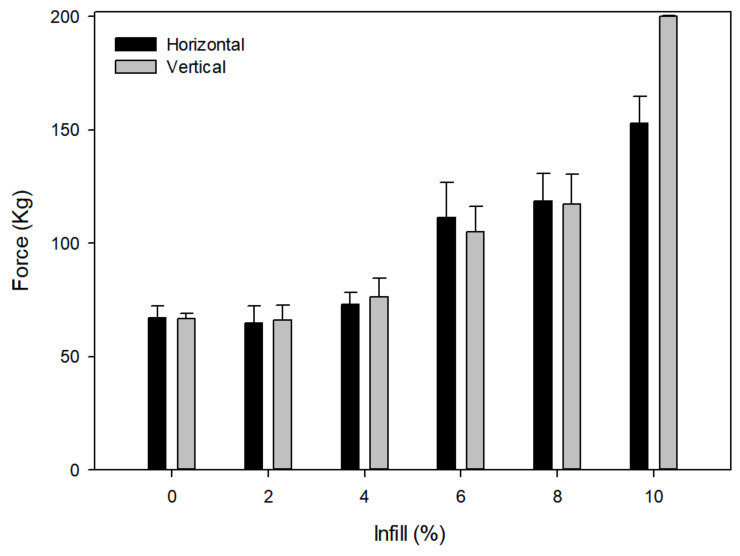
Representation of the force achieved before breaking point. Test of the St PLA 3D constructs in horizontal and vertical position for each infill were assessed (*n* = 5).

It has been demonstrated that infill percentage modulates the dissolution profile. Goyanes et al. studied the release of fluorescein in tablets with different infills and found that the lower the filling of the tablet, the higher the percentage of fluorescein released [37]. Other studies also support these results [26,38]. As can be seen in Figure 5, none of the infills below 10% allowed maximal strength. The 10% fill was selected to carry out the diffusion study, since it was the first one to resist to the overloading point under vertical forces. Additionally, significant differences (*p* < 0.05) were found among infills, both vertically and horizontally. As can be observed (Figure 5), increasing the infill increases the resistance to load. 

### 2.4. Release Studies 

In vitro substance release studies from the 3D constructs were performed to characterize how the constructs were able to release their load when placed in contact with aqueous media. Table 4 shows the equations obtained by fitting the different kinetic models to methylene blue release results from the 3D constructs.

After evaluating the statistical parameters r and SS, the Krosmeyer-Peppas equation best describes the release of methylene blue through the micropores of the 3D construct. In this model the release exponent n indicates the mechanisms to describe how the active compound is released from the matrix; an exponent *n* ≤ 0.5 is characteristic for Fickian diffusion release [39] which is the case in our model. 

**Figure 6:**
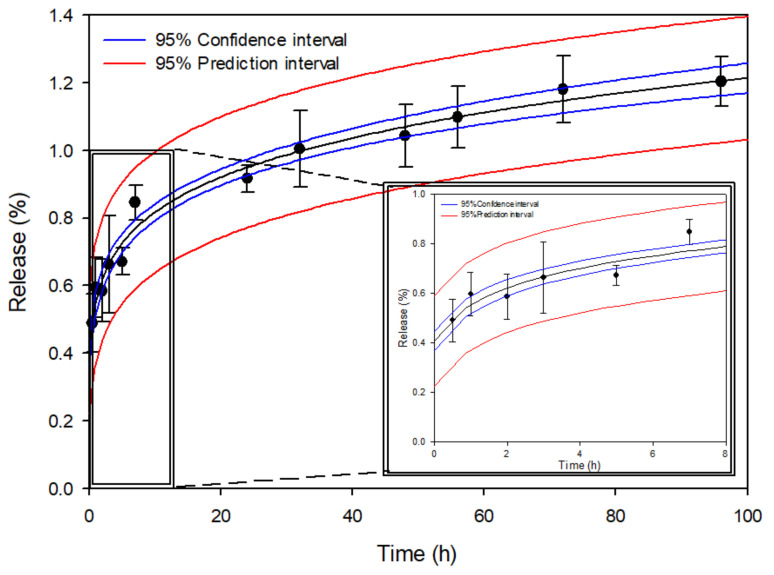
Percentage of methylene blue released from the 3D constructs (model VI) during 96 h, (*n* = 5, mean ± sd).

The release profile of 3D constructs from the insert is shown in Figure 6 which shows that the percentage of methylene blue released from the insert was 1.2% in a 96 h period. Although the conditions of this assay are far from being realistic because the test is performed in an excess of water and with constant stirring, the results obtained show that methylene blue is released from the constructs.

The release of methylene blue from the 3D construct produces a rapid initial increase in the concentration of methylene blue in the medium, probably because the methylene blue from the outermost layers is released rapidly, then stabilizes over time as the methylene blue it is released from the deeper layers of the 3D construct. During the first 7 h the percentage of methylene blue released from the construct was 0.85%, while a 0.35% was further released from the construct until completing the total 96 h (Figure 6). It could be that the release is low due to the exchange of the solvent in the interior of the cylinder with the external media through a system with a relatively low porosity.

**Figure 7:**
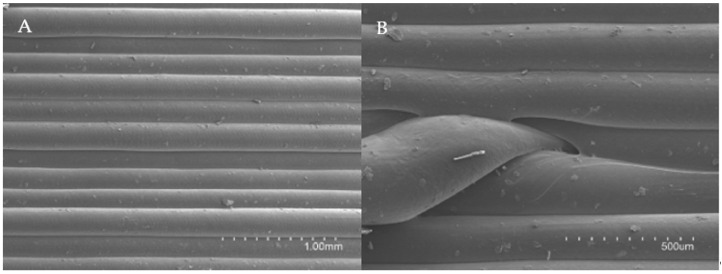
Electron microscope images of the different layers of the printed spacers at 40× magnification (**A**) and micropores formed in each layer at 90× magnification (**B**).

Figure 7 shows the arrangement of the layers as viewed with SEM at 40× (7A) and the micropores formed in each layer at 90× magnification (7B). These images were used to measure the micropores dimensions (Figure 7).

**Figure 8:**
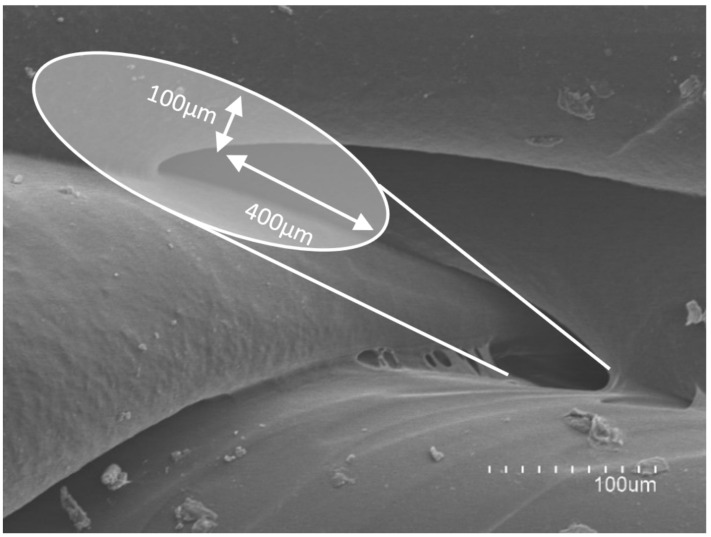
Electron microscope images of the different layers of the printed 3D constructs at 300× magnification.

The micropores size was calculated as the area of the ellipse (A = a·b·π) that most closely fitted its shape (Figure 8). The smallest radius (a) of the ellipse measured 100 µm, and the largest (b) 400 µm.

The total micropores area (PA) was calculated by multiplying the area of each micropore (A) by the number of layers (n) of the cylinder, thus obtaining the total micropores surface. The surface area of each printed cylinder is 4239 mm^2^ and the total porosity surface was deemed to be 19.47 mm^2^. Therefore, as a percentage, the porosity of the cylinders would be approximately 0.46% of the total surface. With the layer height, the printing speed and the number of layers, this porosity surface could be modified, if necessary and the release of a drug thus controlled to achieve its therapeutical objective. These results confirm that 3D FDM printing may allow manufacturing of spacers with incorporated delayed-release drugs, and they would also allow the possibility of adapting the release profile according to the characteristics of the infection, thus personalizing the treatment.

## 3. Materials and Methods 

### 3.1. Materials

The polymers used in the work were PLA, PP, ABS and PET-G and in all cases, the diameter of the filament was 1.75 mm. Extrusion temperatures and printer bed temperatures were set according to the manufacturer’s recommendations (Table 5). Throughout the experiments two types of PLA were used: standard PLA (St PLA) and high temperature PLA (Ht PLA) because as their different properties may affect their mechanical properties. Ht PLA is characterized by an increased thermal stability compared to the St PLA. The softening temperature is slightly above 90 °C, approximately 20 °C higher than with standard PLA. According to the manufacturers, Ht PLA is less brittle or fragile than the Standard PLA and it is characterized for its elevated toughness and stiffness.

### 3.2. Design and 3D Printing

**Figure 9:**
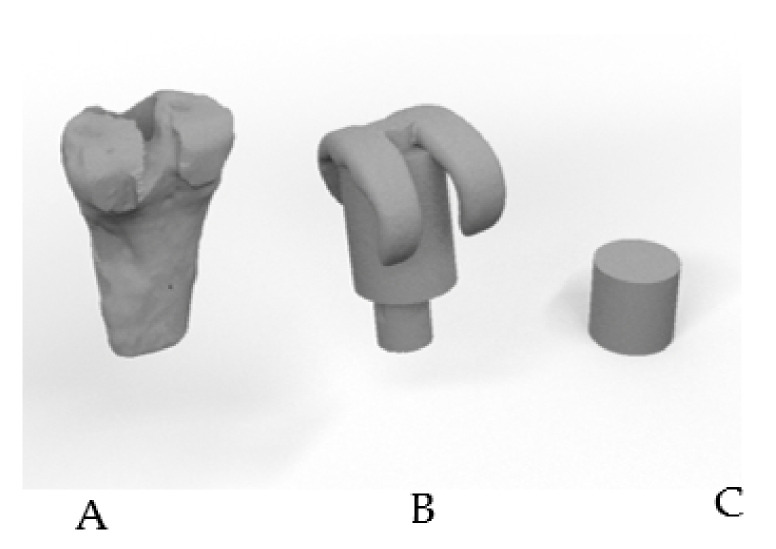
Steps to achieve a simplified volume to study: digitalized femur (**A**), spacer (**B**) and test construct designed (**C**) with the program “Rhinoceros 3D”.

Several constructs were made to select the most appropriate internal design for a spacer allowing the release of the drug while taking into account the resistance that a prosthesis would need. In Figure 9 we can observe the steps taken to transform the femur into a geometric body to evaluate the different properties. The diameter of human femurs from males (33.29 ± 1.55 mm) and females (28.48 ± 0.57 mm) from our University Anatomy Department were measured using a caliber at different points of the distal metaphysis and were used to design a cylinder as the closest regular volume to a real femur. The volume of the cylinder is 23.12 cm^3^ but both the wall thickness and the infill percentage were taken into account to calculate the internal volume available. Digital designs of the cylinder were produced using the program “Rhinoceros 3D” and then printed to further explore both resistance and diffusion.

The 3D printer used was “Flashforge Creator Pro” with a layer resolution of 100–500 microns. The maximal printing volume of the printer has the following dimensions: a base of 227 mm × 148 mm and a height of 150 mm and an extruder with a nozzle diameter of 0.4 mm.

In Table 6 we can observe the parameters used for every material for 3D printing. For each material, the manufacturer’s recommended temperature was used (Table 5).

### 3.3. Physical Characterizations

**Figure 10:**
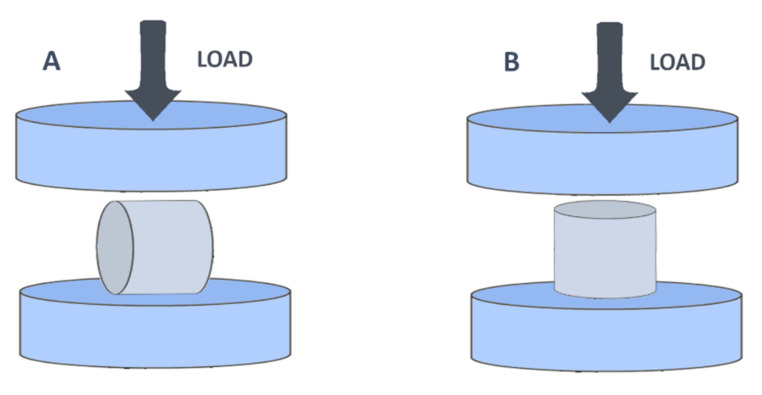
Position of the load force in both horizontal (**A**) and vertical (**B**) positions of the cylinder.

To evaluate the resistance of 3D printed constructs for each material, a compression test was carried out. Ten samples (cylinders) of each material were analyzed. Five samples were used to carry out the study of resistance in a vertical position (compression force applied to the bases of the cylinders) and 5 samples for a horizontal study of resistance (Figure 10).

The compression study of the cylinders was carried out using a computerized-controlled mechanical rack single-track dynamometer (Model Conus 850-I of Instruments J.BOT S.A, Barcelona, Spain). It has a maximum factory capacity of 2 KN. Because some of the cylinders resisted the maximum load, a second dynamometer was used with a higher load limit to complete the study. The compression tests of the remaining cylinders were carried out on a computerized-controlled Zwick/Roell Z005 universal machine (Barcelona, Spain) equipped with a load cell with a load capacity of 5 KN. The test was controlled by a test machine crosshead displacement of 0.005 mm/s. This dynamometer was not used for every cylinder as it was not located in our laboratory and had to be outsourced.

Two measurements were taken with the dynamometer: the load applied at breaking point (Kg) and the compression (mm) obtained prior to breakage. The breaking point was established at a force switching threshold of 5%, precharge at 0.01 MPa, compression speed at 1 mm/min, test speed 10 mm/min and a maximum deformation in compression of 15% of its original dimensions.

The percentage of final compression was calculated according to Equation (1), where % *Cl* is the compression percentage, *Fc* is the final compression and *Ic* is the initial compression:

(1) $$\%\;Cl=\left(\frac{Fc-Ic}{Ic}\right)*100$$

To study how different environmental conditions affect resistance and deformation of the 3D constructs, the printed cylinders were evaluated under different conditions. They were studied at room temperature and ambient humidity, room temperature but submerged in water, at body temperature (37 °C) and ambient humidity, and at body temperature (37 °C) but submerged in water.

Scanning Electron Microscopy (SEM) was used to check the surface morphology of the cylinders using a HITACHI S-4800 Scanning Electron Microscope with Field Emission Gun (FEG) with a resolution of 1.4 nm at 1 KV RX Bruke detector (accelerative voltage 5 KV). Samples of the cylinders were taken and then placed on a SEM sample holder using graphite-impregnated adhesive conductive black carbon tape. The sample was then coated with platinum and visualized by SEM at ×40, ×90 and ×300 magnifications.

### 3.4. In Vitro Release Studies

To study the release of substances through the micropores of the 3D printed constructs, samples of the chosen design (Std PLA, design VI, see Table 1) were printed and filled with 15 mL of a 1 mg/mL solution of methylene blue as a model drug. Just before the top layer was printed, printing was paused, the cylinders were filled with the methylene blue solution and printing was resumed, leaving the solution inside the cylinder.

**Figure 11:**
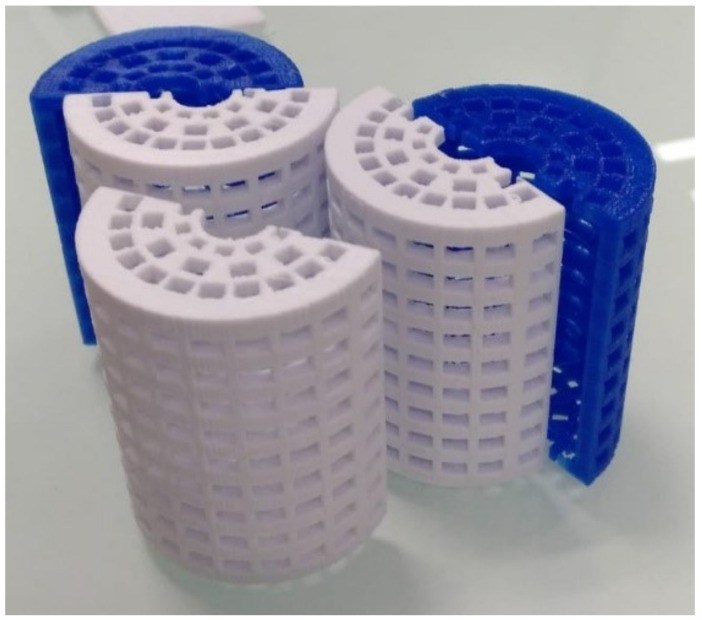
Cages designed with “Rhinoceros 3D” and printed with “Flashforge Creator Pro” to maintain the constructs submerged in the PBS solution in the Dissolution testing equipment. Once the construct was placed between. the two half-cages were weld together with PLA printed clips.

Model drug release was tested using a Dissolution Tester equipment (Erweka DT-80 Series). The vessels were filled with 200 mL of Phosphate Buffer Solution (PBS) at pH 7.4. To prevent the 3D printed constructs from floating in the solution, they were placed inside specially made cages, which were designed with the “Rhinoceros 3D” program and printed with “Flashforge Creator Pro” (Figure 11). The 3D-printed constructs held in the cages were immersed in PBS at 37 ± 0.5 °C and stirred at 25 rpm to simulate extracellular physiological conditions. 

One-ml samples were taken at set intervals 0.5, 1, 2, 4, 6, 24, 30, 48, 54, 72, 78 and 96 h. Immediately after each sample was taken 1 mL of fresh PBS solution was added to the corresponding vessel. The amount of methylene blue in the samples was quantified by spectrophotometry at 660 nm.

The mechanism of release from 3D printed constructs was further investigated by fitting zero order, first order, Higuchi and Korsmeyer-Peppas models to the experimental data. 

The zero-order kinetics model, Equation (2), assumes that drug release is constant and can be characterized with *k*_0_, the zero-order release constant:

(2) $$M_{t}=M_{0}+k_{0}\cdot t$$

where *M_t_* and *M*_0_ are the cumulative amounts of drug release at time t and initial time respectively.

First-order kinetics model corresponds to the following equation:

(3) $$M_{t}=M_{\infty }\cdot \left(1-e^{-k_{1}\cdot t}\right)$$

where *M*_∞_ is the cumulative amount of drug release at infinite time and *k*_1_ is the first-order release constant. 

In the Higuchi model, Equation (4):

(4) $$M_{t}=M_{0}+k_{H}\cdot t^{0.5}$$

*k_H_* corresponds to the release rate constant and reflects the design variables of the system.

The Equation (5) corresponds to Korsmeyer-Peppas model:

(5) $$M_{t}=k_{KP}\cdot t^{n}$$

in which *k_KP_* represents a rate constant incorporating structural and geometric characteristics of the device, and n is the release exponent that provides information of the transport mechanism. 

The curve fittings were performed by non-linear regression, minimizing the sum of the squared residuals. Fitting was performed using the complement DDsolver in Excel (Microsoft) [40]. Statistical parameters used to select the model were r^2^ and the sum of squared residuals (SS) [39].

### 3.5. Statistical Analysis

Results were expressed as mean ± standard deviation (*n* = 5). Nonparametric tests were used to determine statistically significant differences between the experimental groups by means of the Kruskal–Wallis test with post hoc analysis using the Mann–Whitney test applying the Bonferroni correction for the multiple comparisons performed. The confidence level was established at 95%. SPSS 27.0 (IBM) was used.

## 4. Conclusions

The FDM 3D printing technique was evaluated as a spacer creation tool for the development of controlled release systems for drugs. In particular, the release of methylene blue through the micropores created between the layers of cylindrical patterns was evaluated. Different designs and different materials were analysed to obtain a cylinder with suitable properties to be used in in vitro release studies.

Based on our studies, an appropriate material to further this research could be St PLA, which when used in combination with a Flashforge Creator Pro 3D printer, initially demonstrated the possibility of printing cylindrical structures with micropores and interconnected hollow chambers capable of releasing substances contained in the inner hollow reservoir. St PLA cylinders were printed with 10% infill whose physical-technological characteristics, morphological changes after interaction with water and body temperature were considered to be acceptable. The cylinders turned out to have enough resistance to hold 500 kg, opening the opportunity to continue this line of research in the hope of designing personalized prosthetic spacers containing antibiotics to fight in situ infection-causing microbes.

## Figures and Tables

**Table 1:** Digital representation with “FlashPrint” of different spacers design iterations and results obtained with them.

Design Iterations	Description/Comments	
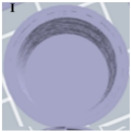	Design I was created with two external layers and a solid thick wall (100% infill). There was an internal chamber where the substance was loaded.	
	Methylene blue was not released in the 3D constructs corresponding to the design I. This could be due to the number of solid layers between the internal chamber and the exterior.	
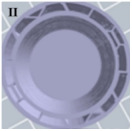	Design II was created with two external layers and a wall with infill of 20% and an internal chamber. The infill of the wall was created with a hexagonal pattern in order to improve resistance.	
	In order to facilitate drug release in design II, the wall of the cylinder was printed with an infill of 20% instead of solid, but still methylene blue was not released.	
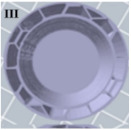	Design III maintained both the internal chamber and the 20% infill of the wall, as well as the hexagonal pattern, but was created with only one external layer in order to improve the release.	
	Even though the wall thickness was reduced, methylene blue was not released. It seemed that having to overcome both the internal wall of the chamber and the external wall was stopping the release.	
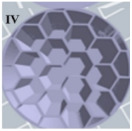	In order to achieve release the internal chamber was removed and instead a number of hexagonal chambers were created with 20% infill using the hexagonal pattern.	
	The result was a massive release of methylene blue as one external layer was proven to be insufficient without the internal chamber and the release was instantaneous. However, after the initial release very little methylene blue was being released.	
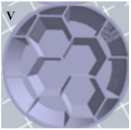	To avoid the first burst of release two external layers were printed instead of one and to increase the overall release infill was reduced to 10% using the hexagonal pattern.	
	Release was inconsistent and erratic and although some diffusion was observed it was considered to be insufficient. This might be due to de hexagonal pattern creating separated volumes throughout the cylinder preventing a homogeneous internal flow between the chambers.	
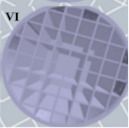	In design VI the hexagonal pattern was substituted for a crisscrossed overlapping-beams (image on the right), which allows for a communicated internal space throughout the cylinder. The infill percentage was kept at 10% and it was printed with two external layers.	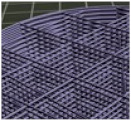
	Methylene blue was released in a sustained manner over time so further research was conducted using design VI.	

**Table 2:** Values of breaking load, compression and percentage of compression of the constructs in vertical position for each material assessed (*n* = 5).

Material	Breaking Load Mean (Kg)	Compression Mean (mm)	Compression (%)
ABS	338.76 ± 31.34	1.94 ± 0.16	6.48 ± 0.53
PET-G	339.83 ± 18.13	2.63 ± 0.26	8.77 ± 0.88
PP	114.04 ± 17.00	2.84 ± 0.21	9.48 ± 0.70
Ht PLA	184.06 ± 42.55	1.10 ± 0.17	3.68 ± 0.56
St PLA	500.00 ± 2.31	0.93 ± 0.17	3.08 ± 0.57

**Table 3:** Values of breaking load, compression and percentage of compression of the spacers in horizontal position for each material assessed (*n* = 5).

Material	Breaking Load Mean (Kg)	Compression Mean (mm)	Compression (%)
ABS	108.77 ± 10.35	2.40 ± 0.42	8.01 ± 1.42
PET-G	102.39 ± 9.04	3.93 ± 0.90	13.12 ± 2.99
PP	41.91 ± 17.64	4.42 ± 0.03	14.74 ± 0.09
Ht PLA	39.90 ± 6.11	1.07 ± 0.29	3.58 ± 0.98
St PLA	156.21 ± 9.57	1.80 ± 0.61	6.02 ± 2.03

**Table 4:** Mathematical model fitting results of methylene blue release, M is amount released (%).

Model Equations	Methylene Blue Kinetics
Zero order	M (%) = 0.651 (±0.022) + 0.007 (±0.0005) t (r = 0.920, SS = 0.076)
First order	M (%) = 1.029 (± 0.031) (1 − e ^−0.436 (±0.058)t^) (r = 0.824, SS = 0.311)
Higuchi	M (%) = 0.523 (±0.091) + 0.076 (± 0.012) t ^0.5^(r = 0.975, SS = 0.034)
Korsmeyer-Peppas	M (%) = 0.551 (±0.018) t ^0.171 (±0.009)^ (r = 0.986, SS = 0.020)

**Table 5:** Characteristics of the different materials obtained from the manufacturer’s information and recommendations for 3D printing.

Polymer	Provider	Density (g/cm^3^)	Extrusion Temperature (°C)	Printer Bed Temperature (°C)
Standard Polylactic Acid (St PLA)	BQ^®^ (Huesca, Spain)	1.24	205	50
High Temperature Polylactic Acid (Ht PLA)	Orbi-tech^®^ (Germany)	1.5	220	70
Polypropylene (PP)	León 3D^®^ (León, Spain)	0.9	195	90
Acrylonitrile butadiene styrene (ABS)	León 3D^®^ (León, Spain)	1.04	240	85
Polyethylene terephthalate (PET-G)	León 3D^®^ (León, Spain)	1.27	220	80

**Table 6:** 3D Printing parameters for each material used.

Polymer	Base Printing Speed (mm/s)	Exterior Layer Printing Speed (mm/s)	Layer Heigh (mm)	First Layer Printing Heigh (mm)	First Layer Printing Speed (mm/s)
St PLA	60	30	0.2	0.4	100
Ht PLA	45	20			
PP	40	20			
ABS	60	30			
PET-G	45	20			

## Data Availability

Data are contained within the article.
